# Comparative Study of Polyethylene, Polypropylene, and Polyolefins Silyl Ether-Based Vitrimers

**DOI:** 10.1021/acs.iecr.4c04006

**Published:** 2024-12-13

**Authors:** Subhaprad Ash, Rishi Sharma, Muhammad Rabnawaz

**Affiliations:** a School of Packaging, Michigan State University, East Lansing, Michigan 48824-1223, United States; b Department of Chemistry, Michigan State University, East Lansing, Michigan 48824-1223, United States

## Abstract

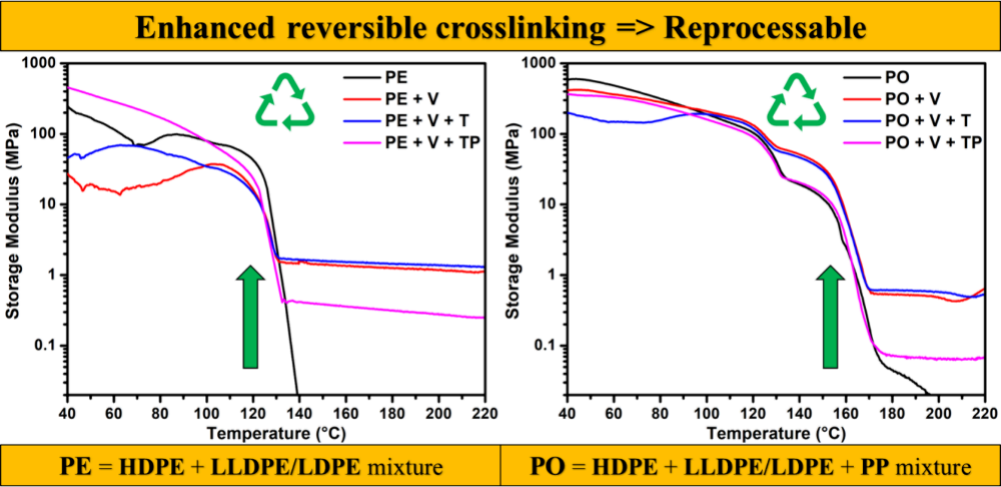

Polyolefins (POs), which constitute over 50% of all plastics, predominantly end up in landfills. To date, there have been no reports on mixtures of PO vitrimers. This study reports the successful synthesis of PO vitrimers from a mixture of 27.7% high-density polyethylene (HDPE), 36.3% linear low-density polyethylene (LLDPE)/low-density polyethylene (LDPE), and 36.0% polypropylene (PP), which is similar to that of Municipal Solid Waste (MSW). This is achieved by using silyl ether-based chemistry, both with and without nitroxides. Additionally, these PO vitrimers are compared with individual vitrimers made of HDPE, LDPE, LLDPE, and PP, as well as vitrimers made from PE blends (comprising HDPE, LLDPE, and LDPE). All vitrimers were prepared via melt extrusion. Their cross-linking density, storage modulus, tensile properties, and reprocessability were assessed. For PO vitrimers, a storage modulus of 0.61 MPa was achieved, indicating a cross-linked network while also maintaining complete melt reprocessability. This study not only provides fundamental insights but also presents a sustainable pathway for recycling PEs and POs into useful materials, hence helping to minimize waste.

## 1.0 Introduction

Polyolefins (POs), composed of polyethylenes (PEs) and polypropylene (PP), are the most widely used thermoplastics and account for nearly 50% of all plastics produced and used today.^1^ However, they are also a significant source of plastic waste.^2^ POs are composed of different types of plastics such as low-density polyethylene (LDPE), linear low-density polyethylene (LLDPE), high-density polyethylene (HDPE)) and polypropylene (PP), but their densities are less than 1 g/cm^3^. Consequently, it is very difficult to separate POs into separate PE and PP. On the other hand, the challenge with using POs without further separation into PE and PP is that they have inferior properties because of their incompatibility.^3^

One promising solution to this problem is the creation vitrimers from POs using thermally reversible cross-links in these systems.^4^ This approach has the potential to revolutionize PO recycling by enhancing their reusability and, thus, reducing the environmental impact of plastic waste. On one hand, physical cross-linking in PE, achieved through ionic interactions and hydrogen bonding, has led to shape memory effects, but at high temperatures, such physical cross-linking is less effective.^5,6^ On the other hand, the area of dynamic chemical cross-linking has been extensively explored and used to create polymers that can flow like vitreous silica above their melting temperature without exhibiting an abrupt drop in viscosity. These special polymers, named vitrimers by Leibler in 2011,^7^ have been the subject of extensive research, particularly with regard to the use of exchange chemistry to fine-tune their properties. Dynamic chemical cross-links provide superior mechanical strength for demanding applications compared to nonbonding interactions. Vitrimers operate via an associative mechanism, where bond formation occurs simultaneously with bond breaking, maintaining the cross-linking density within the system.^8−10^ On the contrary, dissociative mechanisms, such as those obtained through Diels–Alder chemistry, result in a sudden decrease in cross-linking density because bond breaking precedes bond formation.^8−10^ Consequently, numerous studies have focused on vitrimers utilizing associative mechanism chemistries, such as boronic ester metathesis,^11−20^ transesterification,^21−24^ and vinylogous urethane.^5^ However, these vitrimers degrade more rapidly in hydrophilic environments and are prone to hydrolysis, necessitating a thermally and oxidatively stable chemical moiety. Additionally, the high catalyst loading in these systems leads to leaching over time, indicating the need for a catalyst-free fast exchange mechanism.

Recently, we reported that HDPE silyl ether vitrimers showed good thermal stability and a high storage modulus above the melting temperature of the polymer.^25^ There are reports of vitrimers from HDPE, LDPE, and PP employing different exchange chemistries such as dynamic dioxaborolanes^11−20^ and transesterification.^21−24^ Some recent studies have also reported cross-linked PE elastomers that were obtained via dynamic cross-linking.^26,27^ However, no studies have explored silyl ether-based vitrimers for mixed polyethylenes (PEs) combining HDPE and LLDPE/LDPE, or for polyolefins (POs) composed of HDPE, LLDPE/LDPE, and PP. Considering that POs account for an astonishing 62.8% of plastic waste generated, this study focused on exploring new applications for POs in the form of vitrimers.^28^ Therefore, we report for the first time the synthesis of vitrimers made of PEs (HDPE and LLDPE/LDPE), and POs (HDPE, LLDPE/LDPE, and PP) using silyl ether-based chemistry in both the presence and absence of nitroxides. Given that POs in municipal solid waste (MSW) consist of approximately 27.7% HDPE, 36.3% LLDPE/LDPE, and 36.0% PP,^28,29^ we formulated a PO vitrimer to parallel this composition and simulate real-world scenarios. Additionally, PE and PO vitrimers were compared with vitrimers made separately from HDPE, LDPE, LLDPE, and PP.

## 2.0 Experimental Section

### 2.1 Materials

HDPE (DOWLEX IP 1026232) with a melt flow rate (MFR) (190 °C/2.16 kg) of 9*g*/10 min, LDPE (*Petrothene* NA860008) with a MFR (190 °C/2.16 kg) of 24*g*/10 min, LLDPE (DOWLEX 2645G) with a MFR (190 °C/2.16 kg) of 0.9*g*/10 min, PP (SABIC PP 500P) with a MFR (230 °C/2.16 kg) of 3.1*g*/10 min polymeric resins were used in this study. Vinyltrimethoxysilane (VTMS, 98%), dicumyl peroxide (DCP, 98%), 2,2,6,6-tetramethyl-4-piperidinol (T, 98%), and 2,2,6,6-tetramethylpiperidine 1-oxyl (TEMPO, also denoted in this study as TP, 98%) were purchased from Sigma-Aldrich, Milwaukee, WI. Bis[3-(trimethoxysilyl)propyl]amine (BTMSPA, 97%) was purchased from TCI AMERICA, USA. These chemicals were used as received from the commercial suppliers without any further purification.

### 2.2 Synthesis of Polyolefinic Vitrimers

Table 1 lists the materials used in the synthesis of vitrimers and their roles. Table 2 summarizes the compositions of the polyolefinic vitrimers using the above materials. Physical mixing was used to combine all the chemicals, which were then placed into a 15 cc DSM microcompounder (15HT, Xplore Instruments BV, The Netherlands) equipped with twin screws that rotate conically. The reaction proceeded in a molten state in a nitrogen environment while the screws were rotating at 100 rpm. The torque of the system was equilibrated to a constant value after 4 min of residence time for the polymer at 190 °C. Subsequently, the samples were extruded and injection molded at 190 °C using an injection molder IM5.5 #0802, (Xplore Instruments BV, The Netherlands) to produce samples for dynamic mechanical analysis (DMA) analysis, as well as thermal analysis and tensile strength testing. The molten material was injected into the mold at a pressure of 6 bar while the mold’s temperature was kept at 45 °C.

**Table 1:** List of material codes and their functions in the study

**Material**	**Code**	**Function**
High-density polyethylene	HDPE	polymer
Linear low-density polyethylene	LLDPE	polymer
Low-density polyethylene	LDPE	polymer
Polypropylene	PP	polymer
Polyethylenes	PE	polymer mixture of HDPE, LLDPE, and LDPE
Polyolefins	PO	polymer mixture of HDPE, LLDPE, LDPE, and PP
Vinyltrimethoxysilane	V	grafting agent
Dicumyl peroxide	DCP	radical initiator
Bis[3-(trimethoxysilyl)propyl]amine	BTMSPA	silyl ether exchange cross-linker
2,2,6,6-Tetramethyl-4-piperidinol	T	radical scavenger
2,2,6,6-Tetramethylpiperidine 1-oxyl (TEMPO)	TP	radical scavenger

**Table 2:** Formulations (mol %) of materials used to produce PO vitrimers^a^

**Sample**	**HDPE** (mol %)	**LLDPE** (mol %)	**LDPE** (mol %)	**PP** (mol %)	**DCP** (mol %)	**V** (mol**%)**	**BTMSPA** (mol %)	**T** (mol**%)**	**TP** (mol %)
HDPE Series									
HDPE	100.00								
HDPE+V	98.99				0.02	0.49	0.50		
HDPE+V+T	98.96				0.02	0.50	0.50	0.03	
HDPE+V+TP	98.96				0.02	0.50	0.50		0.03
LLDPE Series									
LLDPE		100.00							
LLDPE+V		98.99			0.02	0.49	0.50		
LLDPE+V+T		98.96			0.02	0.50	0.50	0.03	
LLDPE+V+TP		98.96			0.02	0.50	0.50		0.03
LDPE Series									
LDPE			100.00						
LDPE+V			98.99		0.02	0.49	0.50		
LDPE+V+T			98.96		0.02	0.50	0.50	0.03	
LDPE+V+TP			98.96		0.02	0.50	0.50		0.03
PP Series									
PP				100.00					
PP+V				98.99	0.02	0.49	0.50		
PP+V+T				98.96	0.02	0.50	0.50	0.03	
PP+V+TP				98.96	0.02	0.50	0.50		0.03
PE Series (HDPE, LDPE, and LLDPE)									
PE	42.00	29.00	29.00						
PE+V	38.36	26.50	26.50		0.02	0.49	0.50		
PE+V+T	38.36	26.50	26.50		0.02	0.50	0.50	0.03	
PE+V+TP	38.36	26.50	26.50		0.02	0.50	0.50		0.03
PO Series (HDPE, LDPE, LLDPE, and PP)									
PO	26.21	18.07	18.07	25.00					
PO+V	23.93	16.50	16.50	22.81	0.02	0.49	0.50		
PO+V+T	23.93	16.50	16.50	22.81	0.02	0.50	0.50	0.03	
PO+V+TP	23.93	16.50	16.50	22.81	0.02	0.50	0.50		0.03

a HDPE+V+T denotes a vitrimeric sample created from HDPE with 0.5 mol % Vinyltrimethoxysilane (V) and 0.5 mol % silyl ether cross-linker with a nitroxide radical scavenger 2,2,6,6-Tetramethyl-4-piperidinol (T)*.*

### 2.3 Differential Scanning Calorimetry

DSC was performed to investigate the thermal properties of injection-molded samples on TA Instruments Q100, USA. Approximately 8.0 ± 1.0 mg of each sample was analyzed under a continuous nitrogen flow at 100 mL/min. The samples were placed in aluminum DSC pans with lids. The temperature program involved heating the samples from 25 to 200 °C at a rate of 10 °C/min, followed by cooling at the same rate. After cooling, the samples underwent a second heating cycle under identical conditions.

### 2.4 Thermogravimetric Analysis

TGA was performed on the vitrimers using a TA Instruments | Waters Discovery TGA 550, USA instrument. Similar to the DSC procedure, approximately 8.0 ± 1.0 mg of sample was placed in an aluminum pan. The samples were heated from 25 to 600 °C at a rate of 10 °C/min under a continuous nitrogen flow for TGA analysis.

### 2.5 Ultimate Tensile Testing

Ultimate tensile tests were conducted on the samples after they had been conditioned for 48 h at 23 °C. The tests were performed using an Instron 5565P6021, USA machine, following the ASTM D638 standard.^30^ Type V specimens were prepared via injection molding with dimensions of 3.25 mm (width), 3.25 mm (thickness), and 12.5 mm (gauge length). The tests were carried out at an extension rate of 10 mm/min. Five replicates of each sample were tested, and the mean values were reported along with their standard deviation.

### 2.6 Dynamic Mechanic Analysis

DMA tests were performed using an RSA-G2 system (TA Instruments), USA. Rectangular specimens were prepared by injection molding, with dimensions of 12.5 × 6.25 × 3.25 mm. These specimens underwent a temperature sweep at a rate of 2 °C/min, ranging from 40 to 220 °C, at a frequency of 1 Hz and 0.01% strain in tensile mode. For control samples, the analysis was conducted over a temperature range from 40 to 170 °C due to their melt flow behavior. The DMA system was equilibrated at 40 °C for 5 min prior to the analysis of each sample.

### 2.7 Fourier-Transform Infrared Spectroscopy

FT-IR spectroscopy was conducted using a Jasco FTIR-6600, Easton, MD, USA, that was equipped with an ATR Pro-One accessory featuring a 28° Michelson interferometer. Spectra were recorded over a wavelength range of 400–4000 cm^–1^, with the number of scans set to auto mode. The collected signals were processed by using the Spectra Manager software.

### 2.8 Stress-Relaxation Experiments

Stress-relaxation tests were performed using a TA-ARES G2 rheometer, USA, which was equipped with a Rheometric Scientific Oven for precise temperature control (±0.1 K). The tests were conducted with an 8 mm parallel plate geometry and a sample thickness of 0.8 mm. After a 10 min temperature equilibration at each temperature from 140 to 150 °C, in 5 °C increments, a constant strain of 5% was applied, and the stress was monitored over time. Based on prior strain sweep experiments, 5% deformation was confirmed to be within the linear viscoelastic range.

### 2.9 Melt Flow Index

An RR-6MBA Advanced Melt Flow System (Ray-Ran Test Equipment Ltd., Warwickshire, UK) was used to calculate MFI. At 230 °C, the polymers were first allowed to melt for five min before being loaded with 2.16 kg. The weight of the sample that flowed out of the capillary dye over a 10 min period was used to compute the MFI values.

### 2.10 Gel Fraction

A solvent extraction approach was used to measure the gel content in the cross-linked network. For 48 h, a single specimen weighing 200–300 mg was submerged in hot xylene (at 120 °C), with new solvent added after 24 h. The insoluble portion was then dried for 24 h at 80 °C in an oven, and the final weight was obtained. The gel fraction % was calculated as follows:
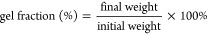
1

## 3.0 Results and Discussion

### 3.1 Synthesis

**Figure 1:**
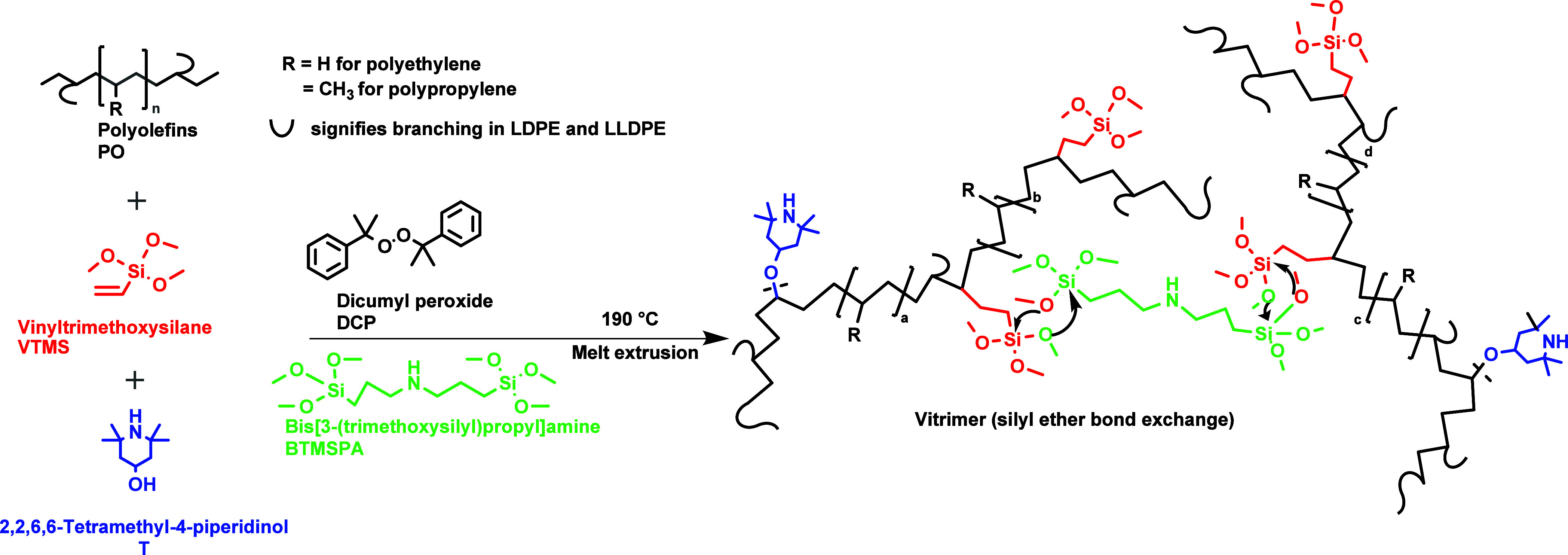
Reaction scheme depicting the creation of PO+V+T vitrimers involving a polyolefin mixture (PO), Vinyltrimethoxysilane (V) and the nitroxide radical scavenger 2,2,6,6-Tetramethyl-4-piperidinol (T). BTMSPA enables cross-linking of silyl ether grafted polymer via silyl ether bond exchange.

The chemistry of PO vitrimers is illustrated in Figure 1. Virgin PO materials (HDPE, LLDPE, LDPE, and PP) were blended with a radical initiator dicumyl peroxide and a silyl ether agent Vinyltrimethoxysilane (V), along with a cross-linker BTMSPA and the radical scavenger 2,2,6,6-Tetramethyl-4-piperidinol (T). The reaction was carried out at 190 °C to ensure all polymers were fully melted, using a microcompounder. As illustrated in Figure 1, V was grafted onto polyolefin polymers and subsequently, the cross-linker BTMSPA facilitated an exchange reaction between silyl ether, thus leading to the formation of reversibly cross-linked vitrimers. Utilizing this method, vitrimers for PE (HDPE, LLDPE/LDPE), as well as individual HDPE, LDPE, LLDPE, and PP vitrimers, were prepared. The formulations for all vitrimers prepared in this study are given in Table 2. 2,2,6,6-Tetramethyl-4-piperidinol (T) was used to improve the efficiency of VTMS grafting onto polymer (PE, PP, and PO) and thus enhance cross-linking density. In particular, T was employed in this investigation because this radical scavenger is known to efficiently bind to the polymer radicals and impede the formation of nonreversible polymer–polymer cross-link structures, as reported in our recent study.^25^

### 3.2 FT-IR Analysis

**Figure 2:**
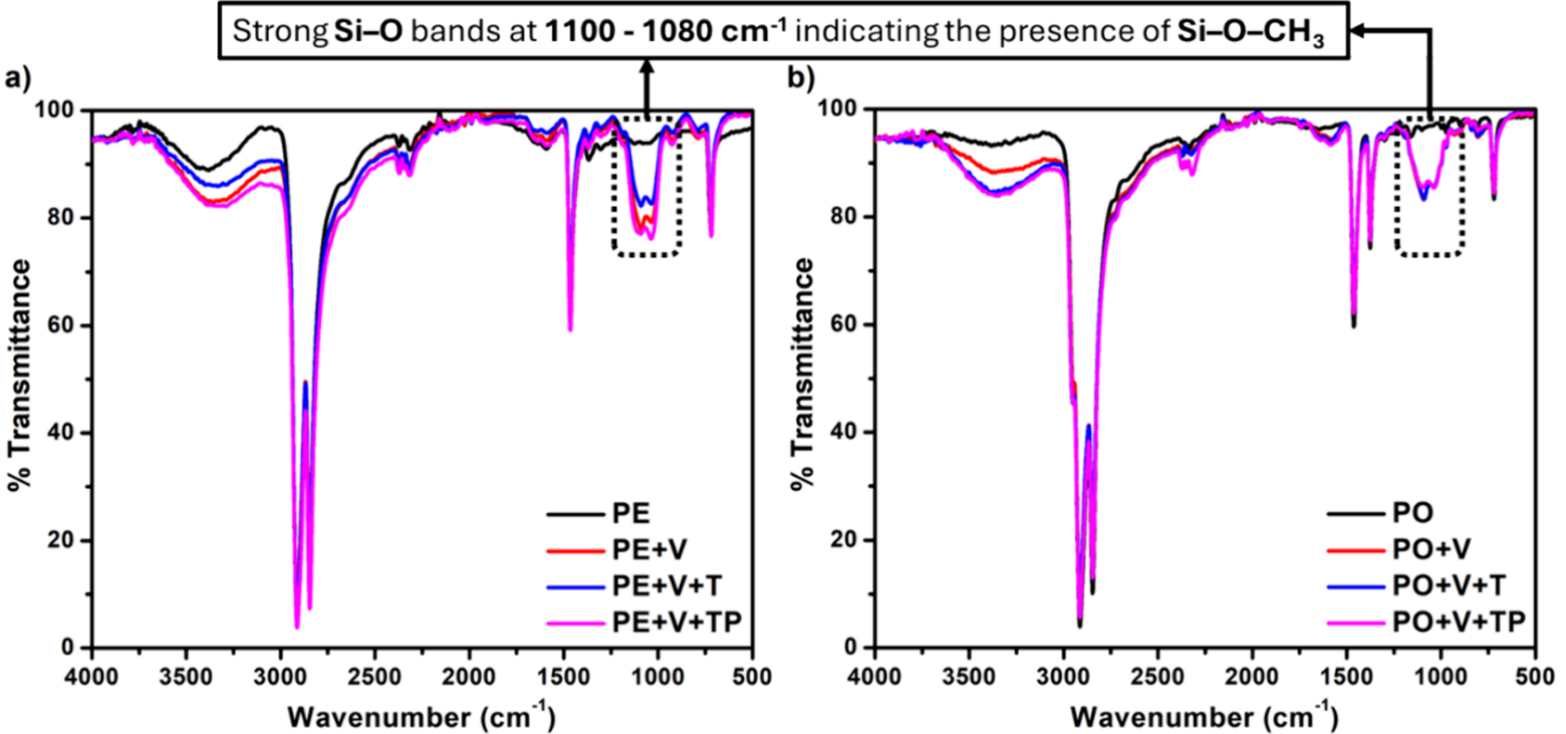
FT-IR spectra for PE (a) and PO (b) vitrimeric systems indicating vinyltrimethoxysilane grafting at 1092 cm^–1^.

FT-IR analyses were used to characterize these vitrimers. The FT-IR spectra of the vitrimers with silyl ether groups revealed several distinctive features. A prominent Si–O stretching band was observed at 1092 cm^–1^, confirming the presence of the silyl ether groups. Characteristic C–H stretching vibrations were identified by strong peaks at 2919 and 2850 cm^–1^, which correspond to the asymmetric and symmetric stretching modes of polyethylene and polypropylene. Additionally, C–H rocking vibrations were evident from the peaks at 730 and 720 cm^–1^. To differentiate between polyolefins having PP in addition to PE, the spectrum of PO exhibited distinctive peaks at 1376 cm^–1^ corresponding to PP and 1462 cm^–1^ corresponding to methylene groups of PP and PE. In contrast, the PE spectrum only showed the 1462 cm^–1^ peak, lacking the 1376 cm^–1^ peak present in PO (Figure 2).

### 3.3 DMA Analysis

**Figure 3:**
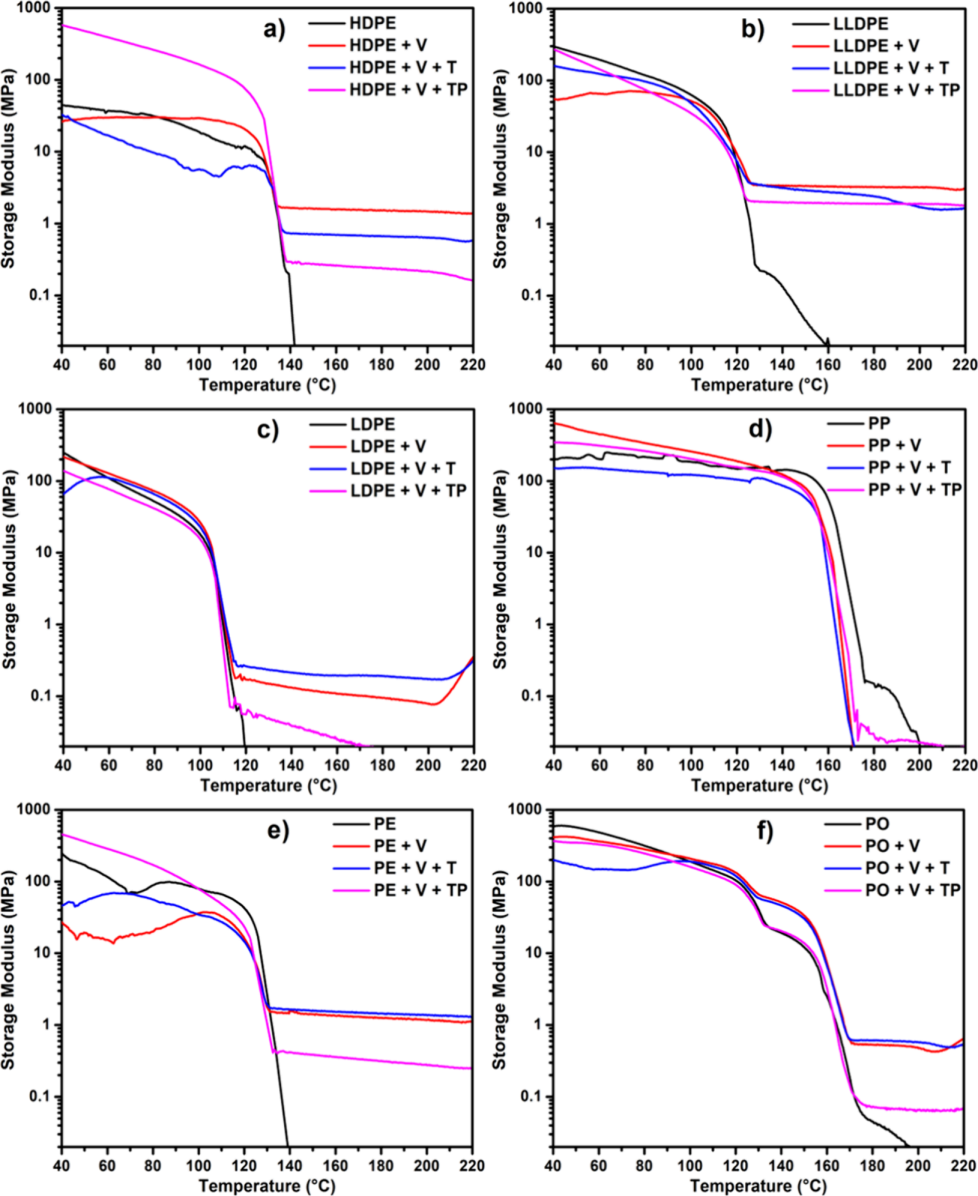
Storage moduli for polymers (black) and their vitrimeric counterparts employing V (red), V+T (blue), and V+TP (pink) for HDPE (a), LLDPE (b), LDPE (c), PP (d), PE (comprised of HDPE, LDPE/LLDPE) (e), and PO (comprised of HDPE, LDPE/LLDPE, PP) (f) vitrimeric systems. The characteristic rubbery plateau above *T*_m_ depicts cross-linking in the polymer. These systems depict very high storage moduli, suggesting that the mixed plastics exhibit good performance.

The storage modulus, a key characteristic of vitrimers, was evaluated via DMA, as shown in Figure 3a–f. In the case of HDPE vitrimers (Figure 3a), control HDPE, which was just thermoplastic, melted as soon as it reached its melting temperature, and its storage modulus dropped to nearly zero at around 140 °C. On the other hand, the HDPE vitrimers continued to show varying storage moduli, with the maximum storage modulus observed at around 1.52 MPa at 180 °C for the HDPE+V system. This finding suggests that in the case of HDPE, the impact of V+T and V+TP was counterproductive as it decreased the storage modulus.

The storage moduli of LLDPE vitrimers were also determined by DMA as shown in Figure 3b. Control LLDPE showed zero storage modulus around 160 °C. However, the LLDPE+V had a strong modulus of 3.28 MPa at 180 °C. Interestingly, the LLDPE vitrimer has a higher storage modulus compared to the HDPE systems.

The most surprising data we obtained were for the LDPE and PP system. The storage modulus for LDPE vitrimers was 0.19 MPa for the V+T system at 180 °C, as shown in Figure 3c. Overall, LDPE performed poorly in terms of vitrimer formation relative to those of HDPE and LLDPE vitrimers. An increase in the storage moduli of LDPE+V and LDPE+V+T was observed beyond 205 °C. This increase may have occurred because the branching in LDPE initially sterically hindered the access of vinyl-grafted LDPE to the cross-linker BTMSPA. At higher temperatures (205 °C), as LDPE became less viscous, the steric hindrance was overcome, exposing more chains to the free radicals and the silyl ether cross-linker present in the system.

**Figure 4:**
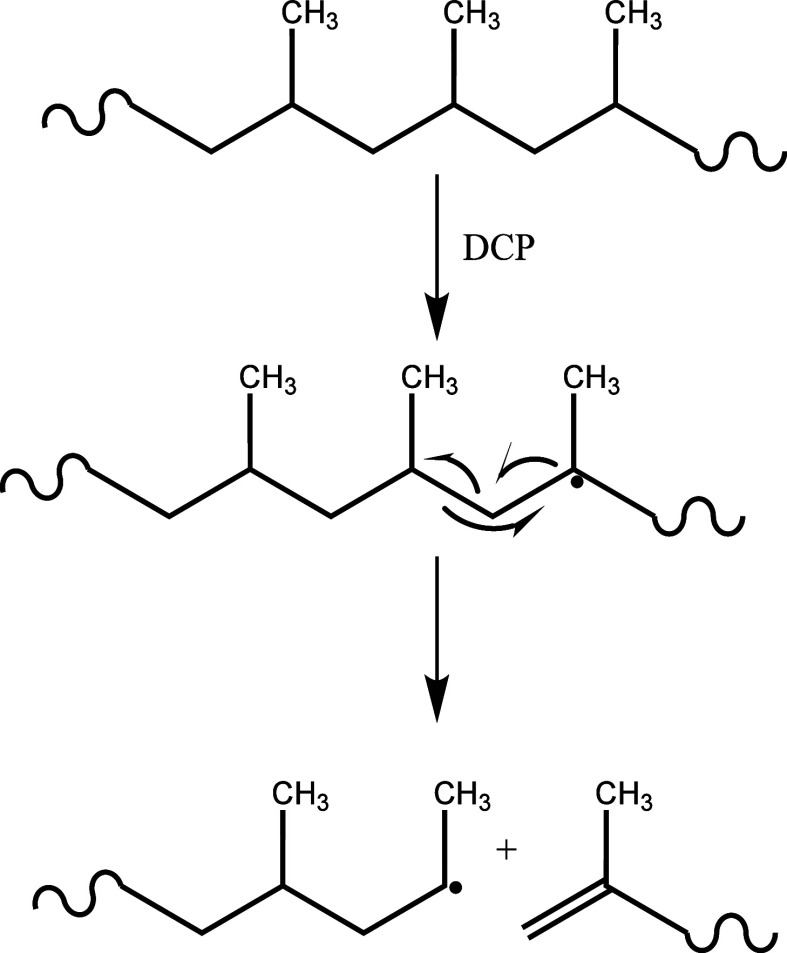
Illustration of PP undergoing potential degradation in the presence of DCP during melt-extrusion.

Figure 3d shows the control PP and its vitrimers. Given PP’s high melting temperature, we observed a complete loss of storage modulus for the control at around 180 °C. A possible reason for this phenomenon could be that V does not effectively react with the PP radical, and PP radicals undergo a rearrangement that leads to the formation of unsaturated fragmented PP rather than V-grafted PP, as shown Figure 4. The degradation of PP was confirmed through melt flow index (MFI) experiments conducted on PP and PP+V+T. Experimentally, PP exhibited an MFI of 4.33 ± 0.55 g/10 min, whereas PP+V+T showed an increased MFI of 7.08 ± 1.25 g/10 min at 230 °C with a load of 2.16 kg. This increase in the MFI for PP+V+T suggests a decrease in molecular weight due to PP degradation during vitrimer formation. Additional evidence for this degradation is observed in the torque measurements during synthesis. While PP experienced a torque of approximately 12 Nm in the extruder, PP+V+T demonstrated a reduced torque of 8 Nm.

The storage modulus data also correlate with gel fraction analysis, as shown in Table S11. Among polyethylenes, the HDPE vitrimer shows a gel fraction of 71.2%, while the LLDPE vitrimer has a gel content of 33.8%. Meanwhile, the PE and PO vitrimers have a gel content of 70.7% and 53.7%, respectively.

Next, we created vitrimers from PE made of HDPE, LDPE, and LLDPE and analyzed their storage moduli. We observed a storage modulus of up to 1.25 MPa at 180 °C, which is lower than what we saw for vitrimers made of HDPE and LLDPE individually. This reduction may be due to the presence of LDPE, which has a much lower storage modulus when used alone. The reason for this could be the same as explained earlier, where we have more efficient grafting onto HDPE and LLDPE, but less efficient grafting onto LDPE.

Subsequently, we determined the storage moduli for PO blends prepared from HDPE, LLDPE, LDPE, and PP. We observed that the storage modulus is 0.53 and 0.61 MPa at 180 °C for PO+V and PO+V+T, respectively. This finding was particularly interesting because we did not see any vitrimer formation from PP alone. From these results, it is possible to form vitrimers from PO blends, but the final performance will depend on the fraction of PP in the blends. The more PP present, the lower the storage modulus will be, likely because PP is less amenable to V grafting and tends to undergo degradation instead.

Table 3 shows the cross-linking densities of vitrimers, recorded based on their storage moduli at 180 °C. The cross-linking density, ν, is measured in units of (× 10^–5^ mol/cm^3^). For the HDPE vitrimers in the HDPE+V system, the cross-linking density reached 13.45 × 10^–5^ mol/cm^3^. In the case of the LLDPE vitrimers, the LLDPE+V system showed a cross-linking density of 28.98 × 10^–5^ mol/cm^3^. This clearly shows that LLDPE is more cross-linked than HDPE. For the LDPE series, the cross-linking density of LDPE+V is 0.84 × 10^–5^ mol/cm3, while for the PP system, the crosslinking density for PP+V is 0 mol/cm^3^. For the PE blends and PO blends, the maximum cross-linking densities were 12.82 × 10^–5^ mol/cm^3^ and 5.39 × 10^–5^ mol/cm^3^, respectively, achieved for PE+V+T and PO+V+T, indicating the role of nitroxide in efficient grafting of V onto PE and PO.

**Table 3:** Cross-linking densities of various vitrimers at 180 °C

**Sample code**	*E***′ at 453.15 K (MPa)**	**Cross-linking density ν (× 10**^**–5**^mol/cm^3^**)**
HDPE Series		
HDPE	0.00	0.00
HDPE+V	1.52	13.45
HDPE+V+T	0.67	5.93
HDPE+V+TP	0.24	2.12
LLDPE Series		
LLDPE	0.00	0.04
LLDPE+V	3.28	28.98
LLDPE+V+T	2.42	21.37
LLDPE+V+TP	1.93	17.08
LDPE Series		
LDPE	0.00	0.00
LDPE+V	0.10	0.84
LDPE+V+T	0.19	1.69
LDPE+V+TP	0.02	0.16
PP Series		
PP	0.13	1.18
PP+V	0.00	0.00
PP+V+T	0.00	0.00
PP+V+TP	0.03	0.22
PE Series		
PE	0.00	0.00
PE+V	1.25	11.09
PE+V+T	1.45	12.82
PE+V+TP	0.32	2.79
PO Series		
PO	0.05	0.40
PO+V	0.53	4.72
PO+V+T	0.61	5.39
PO+V+TP	0.07	0.65

### 3.4 Thermal Analysis (DSC)

**Figure 5:**
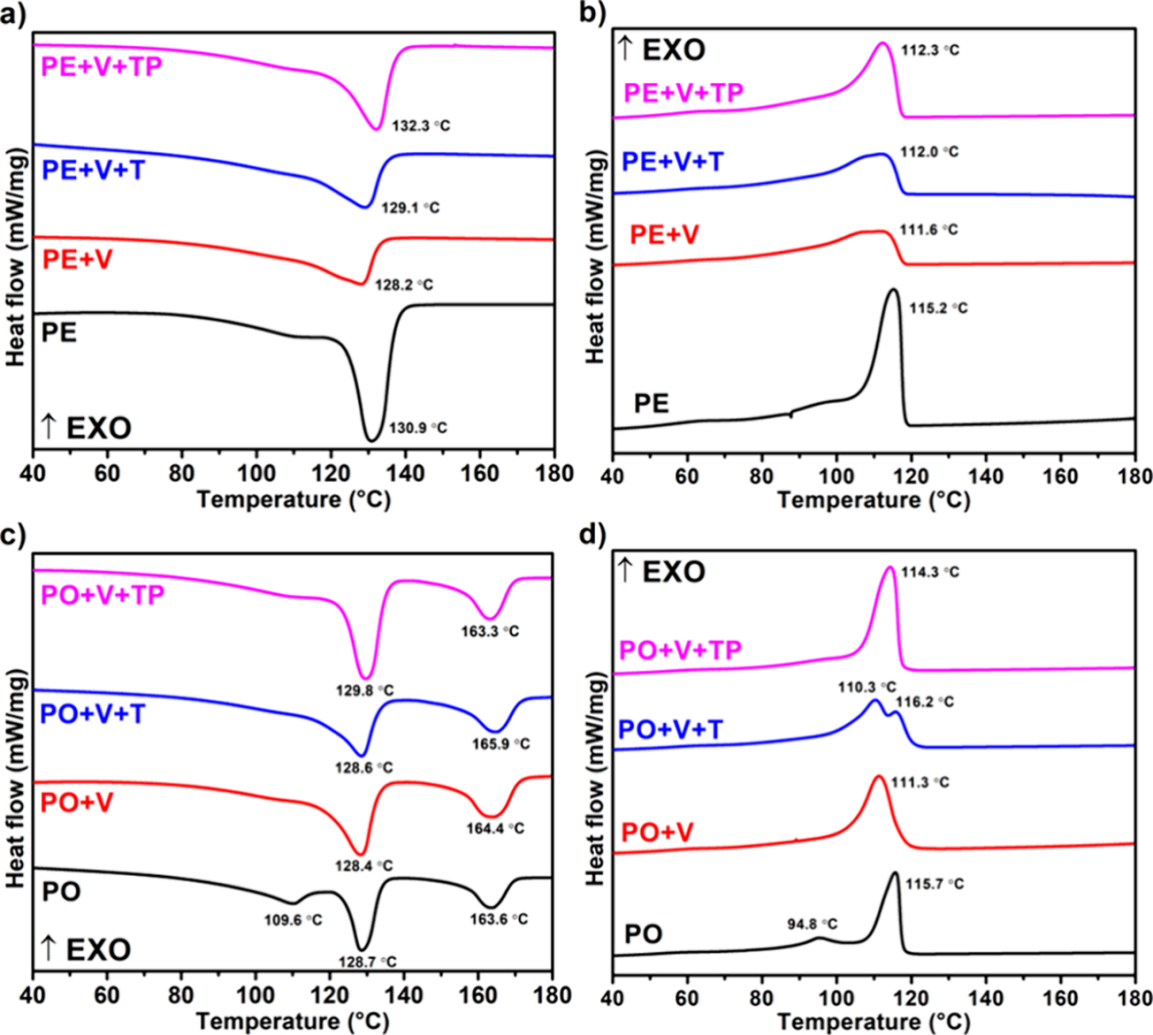
DSC analysis of PE and PO vitrimers. Heating and cooling curves for PE (a, b) and PO (c, d) vitrimers.

Differential scanning calorimetry (DSC) plots for the PE and PO vitrimers are shown in Figure 5 and analysis for individual HDPE, LDPE, LLDPE, and PP vitrimers are shown in the Supporting Information (Tables S1–S4). For the PE blends, a very high melting temperature (*T*_m_)was observed at around 130.9 °C, followed by a smaller shoulder at around 110 °C for LLDPE/LDPE. We also noted that for the PE+V system, where we observed a very good storage modulus, the peak intensity decreased, and the sharp melting peaks turned into broader ones. The broadness of these peaks was roughly proportional to their storage modulus and cross-linking densities; the larger the storage modulus/cross-linking densities, the broader the *T*_m_ peaks. Also, *T*_m_ peak intensities decreased with increasing storage moduli/cross-linking densities. This broadness of *T*_m_ and the reduction in peak intensities are due to the cross-linking in the PE system, which slows down/retard crystallization.

In the case of PO vitrimers, we observe three *T*_m_ peaks for PO at 109.6 °C, 128.7 °C, and 163.6 °C, corresponding to LDPE/LLDPE, HDPE, and PP, respectively. Interestingly, the PP peaks remain intact in terms of intensity and broadness, suggesting that no significant cross-linking had taken place, as evidenced by the storage modulus data. Conversely, the PE peaks become broader and show a decrease in cross-linking density. This behavior clearly suggests that cross-linking occurs in the PE components rather than in PP.

### 3.5 Mechanical Properties

**Figure 6:**
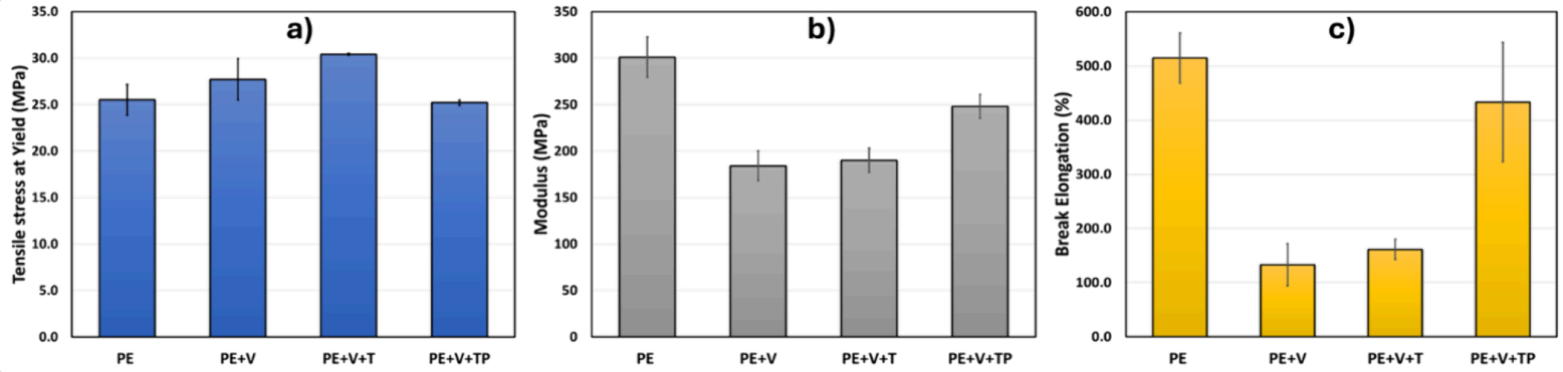
Tensile strength at yield (a), Young’s modulus (b), and elongation at break % (c) for PE vitrimers. We can see the interplay of decreasing crystallinity and increasing cross-linking.

**Figure 7:**
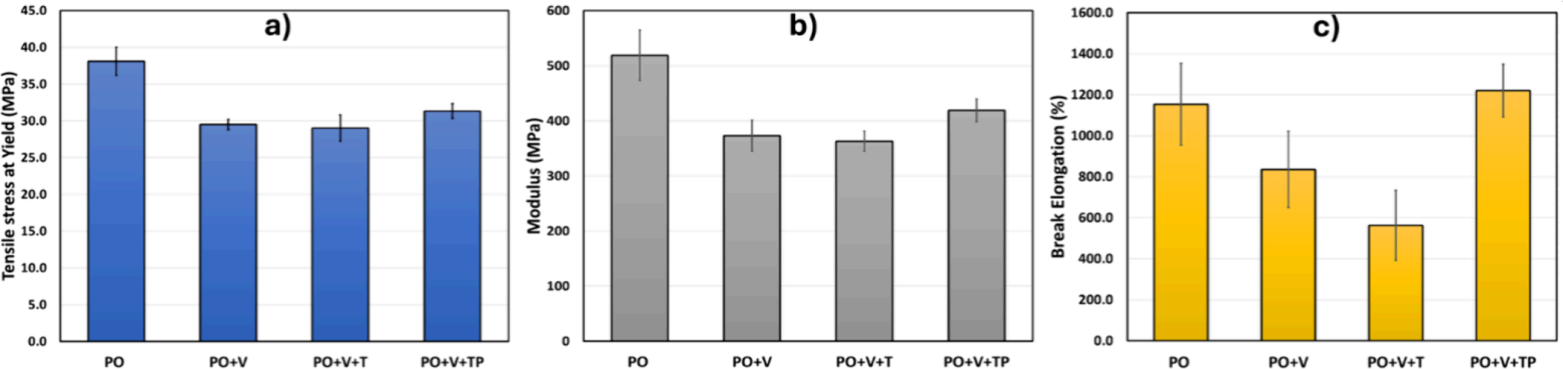
Tensile strength at yield (a), Young’s modulus (b), and elongation at break % (c) for PO vitrimers.

The mechanical properties of both PE and PO samples were analyzed, as shown in Figures 6 and 7, respectively. For the PE samples, which are a blend of HDPE, LDPE, and LLDPE, we observed that the tensile stress at yield was 25.5 MPa for the blend but increased to 30.4 MPa for the PE+V+T system and 27.7 MPa for PE+V. This enhancement can be attributed to the cross-linking in these systems, which requires more energy to deform due to the cross-linking structures. Figure 6b shows that PE exhibited a high Young’s modulus of 301 MPa, but the Young’s modulus for PE+V and PE+V+T were lower. This decrease in modulus might be due to the lower crystallinity of the PE vitrimers, as evidenced by the percentage crystallinity (**χ**_**c**_) (see Table S5 and the heating curve shown in Figure 5a for PE vitrimers). For example, PE has a crystallinity of 51.6% while PE+V and PE+V+T have crystallinity of 41.0% and 36.8% respectively. As expected for thermoplastic PE, one would anticipate much higher elongation because it was not cross-linked, compared to those of PE+V and PE+V+T. Cross-linking of PE resulted in a reduction in elongation, which was an expected behavior. Thus, the mechanical properties observed are in agreement with storage modulus data, where densely cross-linked vitrimers showed a significant decrease in elongation and vice versa.

Figure 7 shows the mechanical properties of the PO and its vitrimers. The PO exhibited a high tensile stress at the yield, but we observed a decrease for the PO vitrimers, as seen in Figure 7a. Young’s moduli of the PO vitrimers were lower than that of the PO itself. This could be attributed to reduced crystallinity in the matrix (In PO systems, we assume PE forms the matrix because of its dominant fraction, and PP becomes a dispersed phase). Additionally, this is consistent with what we noticed in the storage modulus; the higher the storage modulus, the lower the elastic modulus, primarily due to a decrease in crystallinity. While one might expect more cross-linking to yield a higher elastic modulus, in this case, two factors are interplaying: crystallinity and cross-linking. It seems that at room temperature, the temperature at which the tensile properties are tested, crystals in the polymer matrix act as physical cross-linkers, overshadowing the impact of chemical cross-linking on the elastic modulus.

Similar to the decrease in elongation observed in PE vitrimers, the PO system also showed a reduction in elongation at break with increased cross-linking density. However, this was not as pronounced as in the PE systems due to the lower cross-linking density in PO as compared to that of PE (Table 3). Overall, the mechanical properties of PO vitrimers, such as elastic modulus and elongation, correspond to the combined effect of opposing factors: the degree of cross-linking and crystallinity. Apparently, the crystallinity effect determines the modulus because tensile properties were measured at room temperature, and crystalline regions act as physical cross-linkers in amounts much larger than the covalent cross-linking generated in these vitrimers via a silyl ether bond. On the other hand, elongation at break is most likely affected by chemical cross-linking.

In our study, we also investigated the effect of two nitroxides, 2,2,6,6-tetramethyl-4-piperidinol (T) and 2,2,6,6-Tetramethylpiperidine 1-oxyl (TP). TP, marketed as TEMPO, is a stable radical used widely in academia and has now been discontinued by Sigma-Aldrich and Thermo Fisher Scientific. While T is low-cost and readily available, it stands out as the superior nitroxide. In all the HDPE, LLDPE, LDPE, PE, and PO, samples with T outperform samples with TP as observed in Figure 3. Specifically, the PO samples having T exhibited a storage modulus that was 8.7 times higher than those produced using the TP radical scavenger, as illustrated in Table 3. However, PO+V+TP exhibits superior mechanical properties compared to PO+V+T, primarily because of less cross-linking (Figures 6 and 7).

### 3.6 Melt-Reprocessability

**Figure 8:**
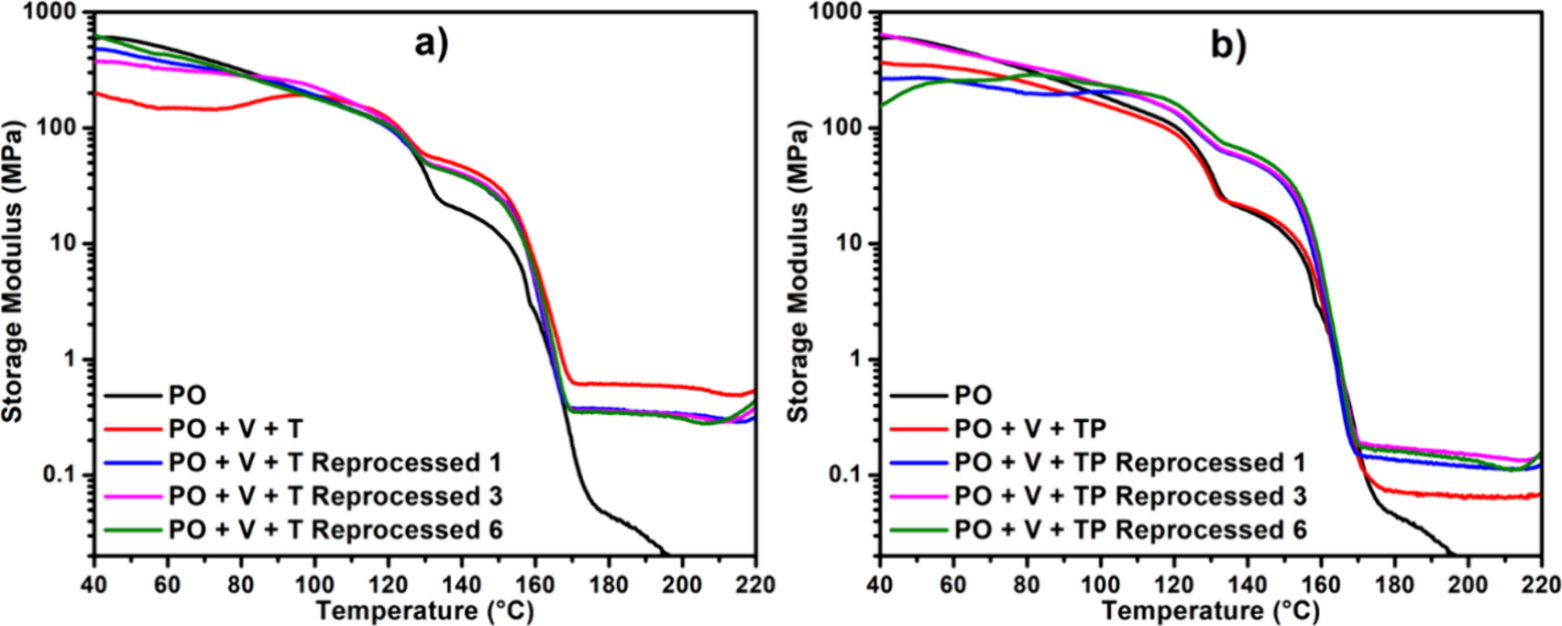
Melt-reprocessing assessment for PO+V+T and PO+V+TP systems showing the storage moduli for 1, 3, and 6 reprocessing cycles. Both the PO+V+T (a) and PO+V+TP (b) systems are reprocessable, but the higher storage modulus for T samples depicts enhanced performance.

Melt-reprocessability of vitrimers is considered as a major benefit of permanently cross-linked polymers. To assess melt-reprocessability, we selected PO+V+T and PO+V+TP systems to observe changes in their storage moduli and appearance. These samples were reprocessed for up to six times and recorded their storage moduli after 0, 1, 3, and 6 processing cycles, as shown in Figure 8a,b. The number 0 denotes the first time we made the vitrimers so that they were not reprocessed at that stage. Figure 8a clearly shows a significant decrease in the storage modulus of PO+V+T as we proceeded from 0 to 1 reprocessing cycles, but no further changes were observed from 1 to 3, and then to 6 reprocessing cycles. In contrast, Figure 8b, where PO+V+TP displayed a different behavior, as the storage modulus was initially lower at 0 cycle but significantly increased after reprocessing cycle 1. From 1 to 3, and then to 6 reprocessing cycles, the modulus remained relatively constant. This could be attributed to the incomplete chemistry in the V+TP system during the first cycle, which underwent further changes, increasing the cross-linking density over time during the reprocessing. This suggests that the chemistry involved in the PO+V+TP system might enhance cross-linking upon repeated reprocessing. The tensile properties of these reprocessed samples, shown in Tables S9 and S10, further indicated that these properties were maintained even after 6 reprocessing cycles.

### 3.7 Stress-Relaxation Behavior

**Figure 9:**
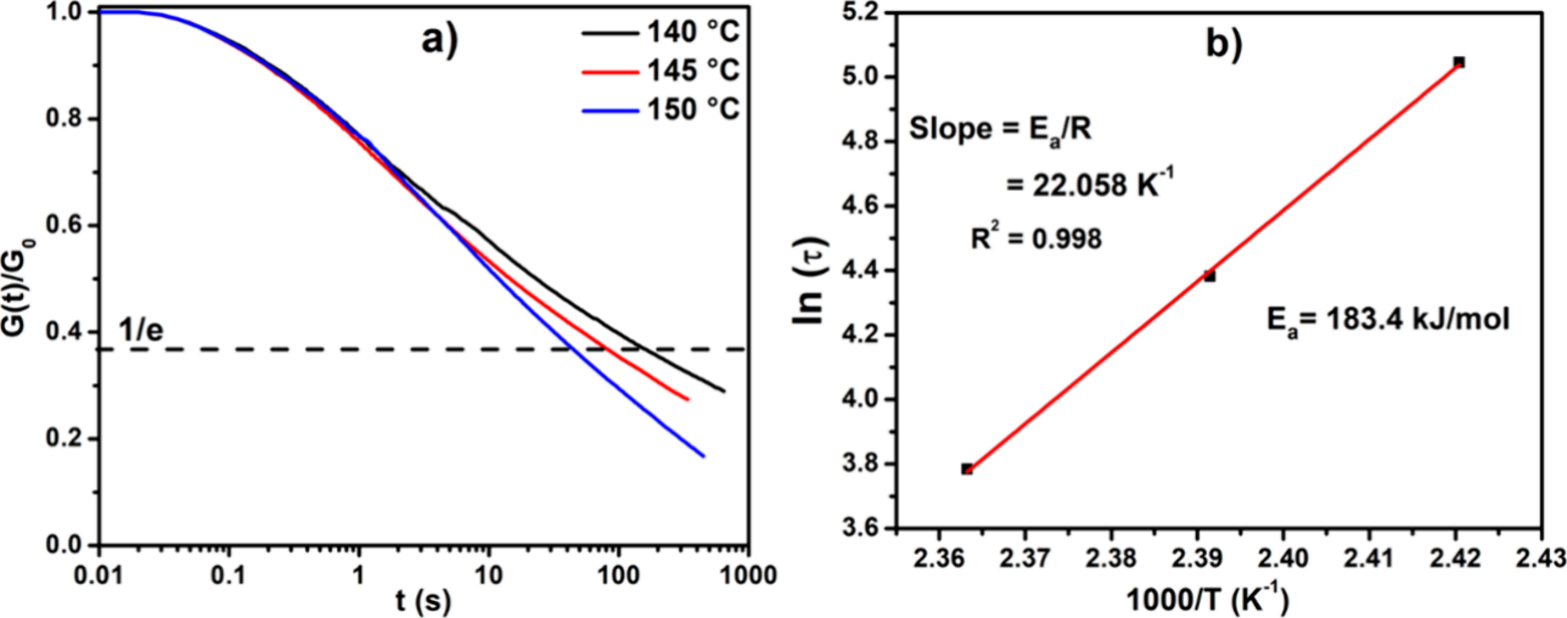
Stress-relaxation plots for the PO+V+TP system.

As a final step, activation energies were calculated for bond exchange in the PO+V+TP system, as shown in Figure 9. This data suggests that the exchange reaction for silyl ether has an activation energy of 183.4 kJ mol^–1^. The activation energy of this silyl ether system is very high compared to those reported in previous studies of silyl ether exchange chemistries. Reports revealed that a silyl ether reaction using camphor sulfonic acid as a catalyst^31^ has an activation energy of 77.8 kJ mol^–1^ and amine-catalyzed^32^ silyl ether exchange has an activation energy of 81 kJ mol^–1^. We attempted to incorporate a silyl ether cross-linker having an amine nitrogen which could enhance the exchange reaction due to the neighboring group effect of nitrogen and eliminate the use of metal catalysts. Although we could not achieve very low activation energy, our study still marks an advantage of eliminating the problem of catalyst leaching while still achieving the exchange chemistry as the sample relaxed to 36.7% in 100 s. Further tuning of the silyl ether cross-linker with diamine systems could possibly decrease the activation energy for the exchange reaction.

### 3.8 TGA

**Figure 10:**
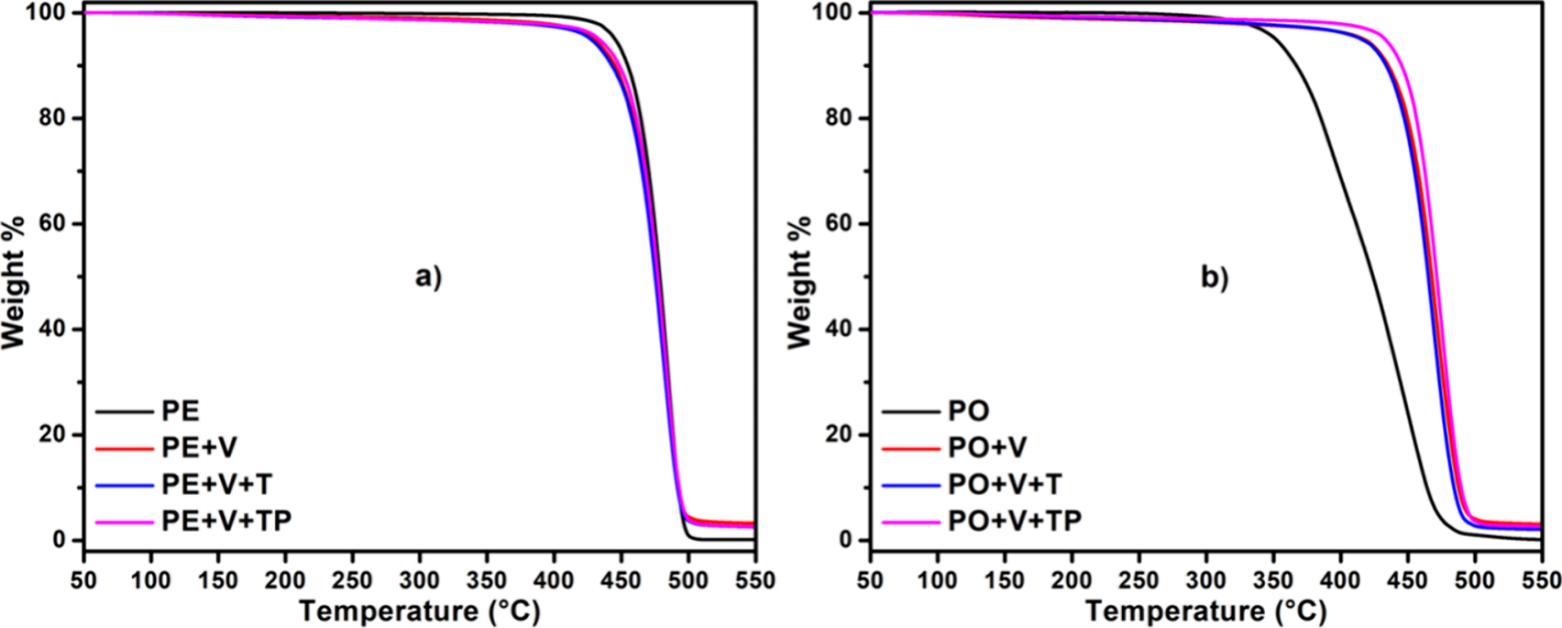
Thermogravimetric analysis showing weight loss with temperature for PE vitrimers (a) and PO vitrimers (b).

Thermogravimetric analysis for PE and PO vitrimers is shown in Figure 10. The vitrimers are thermally stable as indicated by the very high degradation onset temperature, 457 °C and 445 °C respectively, for PE+V+T and PO+V+T. An interesting observation was that PO itself degrades faster (at lower temperatures) than PO vitrimers. For example, the onset of degradation temperature for PO is 350 °C, but that for PO vitrimers is 445 °C, indicating that the vitrimers are more stable. While more studies are needed to determine the exact reason for this, we assume that this could be due to the cross-linked structure tethering PP during degradation to PO vitrimers (slowing down the volatiles formation).

## 4.0 Conclusions

This study is the first of its kind to address a significant knowledge gap by creating PE and PO vitrimers and comparing them with HDPE, LDPE, LLDPE, and PP vitrimers. The rubbery plateau observed beyond the melting temperature (*T*_m_) confirms the presence of cross-linking in all except for the PP samples. This study confirmed that HDPE and LLDPE vitrimers can be prepared with the highest storage modulus. PP does not effectively form any vitrimers, while LDPE forms vitrimers, but with a lower storage modulus. Meanwhile, PE blends form vitrimers but with a storage modulus lower than those of HDPE and LLDPE and higher than that of LDPE. PO can also be transformed into vitrimers, but only the PE fraction is cross-linked, and PP remains mostly un-cross-linked. Additionally, a significant improvement in the storage modulus was observed, facilitated by the use of a more cost-effective radical scavenger, 2,2,6,6-Tetramethyl-4-piperidinol (T), in comparison to the more expensive TEMPO (TP). Furthermore, these vitrimers are melt-reprocessable. Further studies are required to understand the PE and PP compositional impact on the PO vitrimers and testing of non-V grafting agents in PO systems.

## Supporting Information Available

DSC analysis of HDPE, LLDPE, LDPE, PP, PE, and PO vitrimers; tensile properties of PE and PO vitrimers, and reprocessed PO vitrimers; and gel fraction experiments (PDF)

## References

[ref1] Kaminsky W. Polyolefins: 50 Years after Ziegler and Natta I: Polyethylene and Polypropylene; Springer: 2013.

[ref2] Économiques, O. de coopération et de développement Global Plastics Outlook: Economic Drivers, Environmental Impacts and Policy Options; OECD publishing: 2022.

[ref3] Muzata T. S.; Matuana L. M.; Rabnawaz M. Virgin-like High-Density Polyethylene from Recycled Mixed Polyolefins. ACS Appl. Polym. Mater. 2023, 5 (11), 9489–9496. 10.1021/acsapm.3c01955.

[ref4] Khonakdar H. A.; Morshedian J.; Wagenknecht U.; Jafari S. H. An Investigation of Chemical Crosslinking Effect on Properties of High-Density Polyethylene. Polymer 2003, 44 (15), 4301–4309. 10.1016/S0032-3861(03)00363-X.

[ref5] Tellers J.; Canossa S.; Pinalli R.; Soliman M.; Vachon J.; Dalcanale E. Dynamic Cross-Linking of Polyethylene via Sextuple Hydrogen Bonding Array. Macromolecules 2018, 51 (19), 7680–7691. 10.1021/acs.macromol.8b01715.

[ref6] Dolog R.; Weiss R. A. Shape Memory Behavior of a Polyethylene-Based Carboxylate Ionomer. Macromolecules 2013, 46 (19), 7845–7852. 10.1021/ma401631j.

[ref7] Montarnal D.; Capelot M.; Tournilhac F.; Leibler L. Silica-Like Malleable Materials from Permanent Organic Networks. Science 2011, 334 (6058), 965–968. 10.1126/science.1212648. 22096195

[ref8] Scheutz G. M.; Lessard J. J.; Sims M. B.; Sumerlin B. S. Adaptable Crosslinks in Polymeric Materials: Resolving the Intersection of Thermoplastics and Thermosets. J. Am. Chem. Soc. 2019, 141 (41), 16181–16196. 10.1021/jacs.9b07922. 31525287

[ref9] Van Zee N. J.; Nicolaÿ R. Vitrimers: Permanently Crosslinked Polymers with Dynamic Network Topology. Prog. Polym. Sci. 2020, 104, 101233 10.1016/j.progpolymsci.2020.101233.

[ref10] Hammer L.; Van Zee N. J.; Nicolaÿ R. Dually Crosslinked Polymer Networks Incorporating Dynamic Covalent Bonds. Polymers 2021, 13 (3), 396 10.3390/polym13030396. 33513741 PMC7865237

[ref11] Peng L.-M.; Xu Z.; Wang W.-Y.; Zhao X.; Bao R.-Y.; Bai L.; Ke K.; Liu Z.-Y.; Yang M.-B.; Yang W. Leakage-Proof and Malleable Polyethylene Wax Vitrimer Phase Change Materials for Thermal Interface Management. ACS Appl. Energy Mater. 2021, 4 (10), 11173–11182. 10.1021/acsaem.1c02052.

[ref12] Ricarte R. G.; Tournilhac F.; Cloître M.; Leibler L. Linear Viscoelasticity and Flow of Self-Assembled Vitrimers: The Case of a Polyethylene/Dioxaborolane System. Macromolecules 2020, 53 (5), 1852–1866. 10.1021/acs.macromol.9b02415.

[ref13] Ricarte R. G.; Tournilhac F.; Leibler L. Phase Separation and Self-Assembly in Vitrimers: Hierarchical Morphology of Molten and Semicrystalline Polyethylene/Dioxaborolane Maleimide Systems. Macromolecules 2019, 52 (2), 432–443. 10.1021/acs.macromol.8b02144.

[ref14] Maaz M.; Riba-Bremerch A.; Guibert C.; Van Zee N. J.; Nicolaÿ R. Synthesis of Polyethylene Vitrimers in a Single Step: Consequences of Graft Structure, Reactive Extrusion Conditions, and Processing Aids. Macromolecules 2021, 54 (5), 2213–2225. 10.1021/acs.macromol.0c02649.

[ref15] Xiao Y.; Liu P.; Wang W.-J.; Li B.-G. Dynamically Cross-Linked Polyolefin Elastomers with Highly Improved Mechanical and Thermal Performance. Macromolecules 2021, 54 (22), 10381–10387. 10.1021/acs.macromol.1c01249.

[ref16] Wang W.-Y.; Zha X.-J.; Bao R.-Y.; Ke K.; Liu Z.-Y.; Yang M.-B.; Yang W. Vitrimers of Polyolefin Elastomer with Physically Cross-Linked Network. J. Polym. Res. 2021, 28 (6), 210 10.1007/s10965-021-02573-3.

[ref17] Yang F.; Pan L.; Ma Z.; Lou Y.; Li Y.; Li Y. Highly Elastic, Strong, and Reprocessable Cross-Linked Polyolefin Elastomers Enabled by Boronic Ester Bonds. Polym. Chem. 2020, 11 (19), 3285–3295. 10.1039/D0PY00235F.

[ref18] Wang Z.; Gu Y.; Ma M.; Liu Y.; Chen M. Strengthening Polyethylene Thermoplastics through a Dynamic Covalent Networking Additive Based on Alkylboron Chemistry. Macromolecules 2021, 54 (4), 1760–1766. 10.1021/acs.macromol.0c02870.

[ref19] Caffy F.; Nicolaÿ R. Transformation of Polyethylene into a Vitrimer by Nitroxide Radical Coupling of a Bis-Dioxaborolane. Polym. Chem. 2019, 10 (23), 3107–3115. 10.1039/C9PY00253G.

[ref20] Röttger M.; Domenech T.; van der Weegen R.; Breuillac A.; Nicolaÿ R.; Leibler L. High-Performance Vitrimers from Commodity Thermoplastics through Dioxaborolane Metathesis. Science 2017, 356 (6333), 62–65. 10.1126/science.aah5281. 28386008

[ref21] Kar G. P.; Saed M. O.; Terentjev E. M. Scalable Upcycling of Thermoplastic Polyolefins into Vitrimers through Transesterification. J. Mater. Chem. A 2020, 8 (45), 24137–24147. 10.1039/D0TA07339C.

[ref22] Wang S.; Ma S.; Qiu J.; Tian A.; Li Q.; Xu X.; Wang B.; Lu N.; Liu Y.; Zhu J. Upcycling of Post-Consumer Polyolefin Plastics to Covalent Adaptable Networks via in Situ Continuous Extrusion Cross-Linking. Green Chem. 2021, 23 (8), 2931–2937. 10.1039/D0GC04337K.

[ref23] Ji F.; Liu X.; Lin C.; Zhou Y.; Dong L.; Xu S.; Sheng D.; Yang Y. Reprocessable and Recyclable Crosslinked Polyethylene with Triple Shape Memory Effect. Macromol. Mater. Eng. 2019, 304 (3), 1800528 10.1002/mame.201800528.

[ref24] Gao Y.; Liu W.; Zhu S. Reversible Shape Memory Polymer from Semicrystalline Poly(Ethylene-Co-Vinyl Acetate) with Dynamic Covalent Polymer Networks. Macromolecules 2018, 51 (21), 8956–8963. 10.1021/acs.macromol.8b01724.

[ref25] Ash S.; Sharma R.; Shaker M.; Patil S.; Cheng S.; Rabnawaz M. One-Pot Synthesis of Robust Silyl Ether-Based HDPE Vitrimers with Enhanced Performance and Recyclability. Polymer 2024, 308, 127374 10.1016/j.polymer.2024.127374.

[ref26] Xiao Y.; Wang W.-J.; Li B.-G.; Liu P. High-Performance Olefin Thermoplastic Elastomer Based on Dynamically Cross-Linking Crystalline Macromers and Elastic Backbones. Macromolecules 2024, 57 (4), 1788–1794. 10.1021/acs.macromol.3c02372.

[ref27] Chang Y.; Xiao Y.; Sun M.; Gao W.; Zhu L.; Wang Q.; Wang W.-J.; Li B.-G.; Liu P. Oxidative Upcycling of Polyolefin Wastes into the Dynamically Cross-Linked Elastomer. Macromolecules 2024, 57 (21), 9943–9949. 10.1021/acs.macromol.4c01869.

[ref28] Advancing Sustainable Materials Management: 2016 and 2017 Tables and Figures; 2019. https://www.epa.gov/sites/default/files/2019-11/documents/2016_and_2017_facts_and_figures_data_tables_0.pdf.

[ref29] Advancing Sustainable Materials Management: 2017 Fact Sheet; United States Environmental Protection Agency.

[ref30] ASTM International ASTM D638–14, Standard Test Method for Tensile Properties of Plastics; 2015.

[ref31] Tretbar C. A.; Neal J. A.; Guan Z. Direct Silyl Ether Metathesis for Vitrimers with Exceptional Thermal Stability. J. Am. Chem. Soc. 2019, 141 (42), 16595–16599. 10.1021/jacs.9b08876. 31603321

[ref32] Nishimura Y.; Chung J.; Muradyan H.; Guan Z. Silyl Ether as a Robust and Thermally Stable Dynamic Covalent Motif for Malleable Polymer Design. J. Am. Chem. Soc. 2017, 139 (42), 14881–14884. 10.1021/jacs.7b08826. 28991493

