# Supplementary material for: Comparative Study of Polyethylene, Polypropylene, and Polyolefins Silyl Ether-Based Vitrimers
Source: Ind Eng Chem Res. 2024 Dec 13;63(51):22287–97. doi: 10.1021/acs.iecr.4c04006 (PMC11674192; doi:10.1021/acs.iecr.4c04006)
Supplement: Supplementary file 1 — ie4c04006_si_001.pdf [file ie4c04006_si_001.pdf]

Supporting Information

for

Comparative Study of Polyethylene, Polypropylene, and Polyolefins Silyl Ether-Based Vitrimers

Subhaprad Ash[a, b], Rishi Sharma[a], and Muhammad Rabnawaz[a]*

[a] School of Packaging, Michigan State University, East Lansing, MI 48824-1223, USA

[b] Department of Chemistry, Michigan State University, East Lansing, MI 48824-1223, USA

***Corresponding Author:** Muhammad Rabnawaz

Tel.: +1-517-432-4870

Differential scanning calorimetry:

Table S1. Crystallinity and melting point of HDPE vitrimers as obtained from DSC measurements.

Sample	ΔH_m (J/g)	ΔH_c (J/g)	χ_c (%)	T_m (°C)	T_c (°C)
**HDPE**	192.5	205.8	65.6	133.7	112.3
**HDPE+V**	160.9	166.1	54.8	132.2	111.9
**HDPE+V+T**	164.2	174.9	55.9	134.1	113.2
**HDPE+V+TP**	181.9	196.2	62.0	136.6	113.2

Table S2. Crystallinity and melting point of LLDPE vitrimers as obtained from DSC measurements.

Sample	ΔH_m (J/g)	ΔH_c (J/g)	χ_c (%)	T_m (°C)	T_c (°C)
**LLDPE**	135.6	149.5	46.2	124.4	104.2
**LLDPE+V**	105.2	106.4	35.8	118.0	99.6
**LLDPE+V+T**	90.0	101.1	30.6	117.1	97.8
**LLDPE+V+TP**	97.4	105.3	33.2	117.5	98.8

Table S3. Crystallinity and melting point of LDPE vitrimers as obtained from DSC measurements.

Sample	ΔH_m (J/g)	ΔH_c (J/g)	χ_c (%)	T_m (°C)	T_c (°C)
**LDPE**	110.8	116.5	37.7	111.5	97.8
**LDPE+V**	97.6	107.1	33.2	112.3	92.7
**LDPE+V+T**	88.4	102.2	30.1	111.7	91.3
**LDPE+V+TP**	104.9	109.1	35.7	112.2	93.9

Table S4. Crystallinity and melting point of PP vitrimers as obtained from DSC measurements.

Sample	ΔH_m (J/g)	ΔH_c (J/g)	χ_c (%)	T_m (°C)	T_c (°C)
**PP**	107.2	116.1	36.5	165.8	117.2
**PP+V**	152.8	93.3	52.0	**163.9**, 124.5	**112.8**, 103.4
**PP+V+T**	92.2	97.9	31.4	165.9	115.9
**PP+V+TP**	86.7	92.5	29.5	166.3	111.9

Note: Temperature in bold indicates major peak while the second temperature is a small hump.

Table S5. Crystallinity and melting point of PE vitrimers as obtained from DSC measurements.

Sample	ΔH_m (J/g)	ΔH_c (J/g)	χ_c (%)	T_m (°C)	T_c (°C)
**PE**	151.4	160.2	51.6	130.9	115.2
**PE+V**	120.4	138.7	41.0	128.2	**111.6**, 107.86
**PE+V+T**	107.9	124.1	36.8	129.1	**112.0**, 108.04
**PE+V+TP**	126.4	148.4	43.1	132.3	112.3

Note: Temperature in bold indicates major peak while the second temperature is a small hump.

Table S6. Crystallinity and melting point of PO vitrimers as obtained from DSC measurements.

Sample	ΔH_m (J/g)	ΔH_c (J/g)	χ_c (%)	T_m (°C)	T_m (°C)	T_c (°C)
**PO**	103.9	117.4	35.4	**128.7**, 109.6	163.6	**115.7**, 94.8
**PO+V**	103.6	94.6	35.3	128.4	164.4	111.3
**PO+V+T**	98.6	113.7	33.6	128.6	165.9	**110.3**, 116.2
**PO+V+TP**	102.7	129.5	35.0	129.8	163.3	114.3

Note: Temperature in bold indicates major peak while the second temperature is a small hump.

Universal Testing:

Table S7. Tensile properties of PE vitrimers.

Sample code	Tensile stress at Yield (MPa)	Tensile stress at Break (MPa)	Modulus (MPa)	Break Elongation (%)
**PE**	25.5 ± 1.7	19.0 ± 2.7	301 ± 22	514.8 ± 46.3
**PE+V**	27.7 ± 2.2	15.5 ± 6.4	184 ± 16	132.7 ± 39.0
**PE+V+T**	30.4 ± 0.1	21.4 ± 0.5	190 ± 13	161.1 ± 18.6
**PE+V+TP**	25.2 ± 0.3	18.7 ± 2.4	248 ± 13	433.1 ± 110.3

Table S8. Tensile properties of PO vitrimers.

Sample code	Tensile stress at Yield (MPa)	Tensile stress at Break (MPa)	Modulus (MPa)	Break Elongation (%)
**PO**	38.1 ± 1.9	15.5 ± 0.8	519 ± 46	1153.2 ± 199.6
**PO+V**	29.5 ± 0.7	17.5 ± 7.8	373 ± 28	835 ± 186.6
**PO+V+T**	29 ± 1.8	25.7 ± 2.1	363 ± 18	562.2 ± 171.0
**PO+V+TP**	31.3 ± 1.0	32.7 ± 2.5	419 ± 21	1219.9 ± 129.0

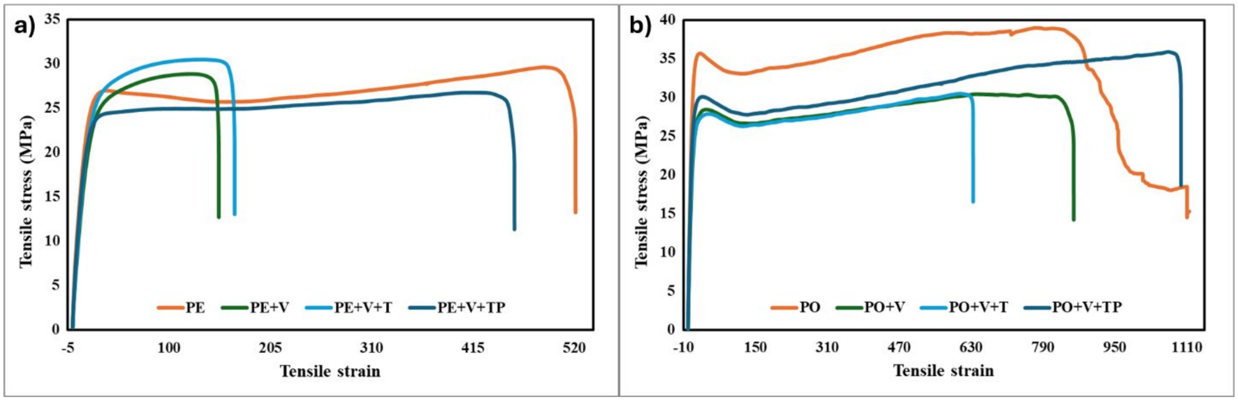

Figure S1. Stress-strain behavior ofPE **(a)** and PO **(b)** systems showing the material behavior during stretching.

Table S9. Tensile properties of reprocessed PO+V+T samples.

Sample code	Tensile stress at Yield (MPa)	Tensile stress at Break (MPa)	Modulus (MPa)	Break Elongation (%)
**PO+V+T**	29.0 ± 1.9	25.7 ± 0.8	363 ± 46	562.2 ± 199.6
**PO+V+T Reprocessed 1**	24.7 ± 0.4	20.3 ± 4.6	348 ± 25	473.2 ± 161.6
**PO+V+T Reprocessed 2**	24.3 ± 0.1	16.5 ± 7.9	362 ± 9	334.4 ± 211.2
**PO+V+T Reprocessed 3**	23.7 ± 1.2	19.9 ± 6.0	350 ± 14	183.7 ± 242.8
**PO+V+T Reprocessed 4**	26.2 ± 1.3	18.8 ± 8.8	350 ± 29	457.1 ± 78.4
**PO+V+T Reprocessed 5**	25.9 ± 0.1	18.4 ± 5.1	374 ± 15	393.5 ± 247.3
**PO+V+T Reprocessed 6**	26.1 ± 0.6	23.5 ± 0.5	405 ± 6.3	236.4 ± 225.9

Note: The terms "Reprocessed 1", "Reprocessed 2", "Reprocessed 3", "Reprocessed 4", "Reprocessed 5", and "Reprocessed 6" refers to samples that had been reprocessed 1, 2, 3, 4, 5, and 6 times, respectively.

Table S10. Tensile properties of reprocessed PO+V+TP samples.

Sample code	Tensile stress at Yield (MPa)	Tensile stress at Break (MPa)	Modulus (MPa)	Break Elongation (%)
**PO+V+TP**	31.3 ± 1.0	32.7 ± 2.5	419 ± 21	1219.9 ± 129.0
**PO+V+TP Reprocessed 1**	27.1 ± 1.7	14.7 ± 4.7	421 ± 7	1011.1 ± 27.5
**PO+V+TP Reprocessed 2**	28.6 ± 0.2	18.0 ± 13.1	437 ± 3	1182.1 ± 30.4
**PO+V+TP Reprocessed 3**	28.4 ± 1.0	23.8 ± 12.9	466 ± 20	1032.2 ± 75.9
**PO+V+TP Reprocessed 4**	29.8 ± 0.7	18.0 ± 12.3	485 ± 8	1047.6 ± 126.3
**PO+V+TP Reprocessed 5**	29.2 ± 1.0	24.0 ± 9.9	499 ± 11	1157.3 ± 125.1
**PO+V+TP Reprocessed 6**	28.1 ± 0.4	15.7 ± 8.3	489 ± 21	1262.9 ± 16.3

Note: The terms "Reprocessed 1", "Reprocessed 2", "Reprocessed 3", "Reprocessed 4", "Reprocessed 5", and "Reprocessed 6" refers to samples that had been reprocessed 1, 2, 3, 4, 5, and 6 times, respectively.

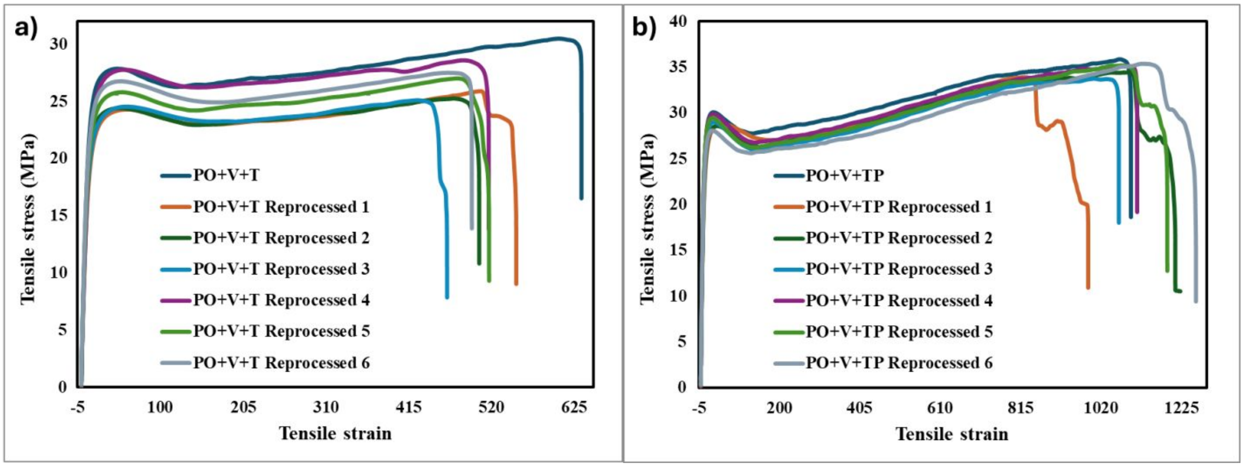

Figure S2. Stress-strain behavior of PO+V+T reprocessed samples **(a)** and PO+V+TP reprocessed samples **(b)** showing good performance after reprocessing.

Gel Fraction:

Table S11. Gel fraction % for vitrimeric samples indicating the amount of crosslinked material in each sample.

Sample	Initial weight (mg)	Final weight (mg)	Gel fraction (%)
**HDPE**	282	0	0.0
**HDPE+V+T**	278	198	71.2
**LLDPE**	259	0	0.0
**LLDPE+V+T**	245	83	33.8
**LDPE**	228	0	0.0
**LDPE+V+T**	219	0	0.0
**PP**	227	0	0.0
**PP+V+T**	214	0	0.0
**PE**	314	0	0.0
**PE+V+T**	297	210	70.7
**PO**	309	0	0.0
**PO+V+T**	311	167	53.7
